# Physical activity is low in obese New Zealand children and adolescents

**DOI:** 10.1038/srep41822

**Published:** 2017-02-03

**Authors:** Yvonne C. Anderson, Lisa E. Wynter, Cameron C. Grant, Joanna M. Stewart, Tami L. Cave, Cervantée E. K. Wild, José G. B. Derraik, Wayne S. Cutfield, Paul L. Hofman

**Affiliations:** 1 Department of Paediatrics, Taranaki District Health Board, New Plymouth, New Zealand; 2 Liggins Institute, University of Auckland, Auckland, New Zealand; 3 Department of Paediatrics: Child and Youth Health, University of Auckland, Auckland, New Zealand; 4 Starship Children’s Health, Auckland District Health Board, Auckland, New Zealand; 5 Centre for Longitudinal Research - He Ara ki Mua, University of Auckland, Auckland, New Zealand; 6 Department of Epidemiology and Biostatistics, University of Auckland, Auckland, New Zealand; 7 A Better Start – National Science Challenge, University of Auckland, Auckland, New Zealand

## Abstract

We aimed to describe physical activity and sedentary behaviour of obese children and adolescents in Taranaki, New Zealand, and to determine how these differ in Māori (indigenous) versus non-indigenous children. Participants (n = 239; 45% Māori, 45% New Zealand European [NZE], 10% other ethnicities) aged 4.8–16.8 years enrolled in a community-based obesity programme from January 2012 to August 2014 who had a body mass index (BMI) ≥ 98^th^ percentile (n = 233) or >91^st^–98^th^ percentile with weight-related comorbidities (n = 6) were assessed. Baseline activity levels were assessed using the children’s physical activity questionnaire (C-PAQ), a fitness test, and ≥3 days of accelerometer wear. Average BMI standard deviation score was 3.09 (SD = 0.60, range 1.52–5.34 SDS). Reported median daily activity was 80 minutes (IQR = 88). Although 44% of the cohort met the national recommended screen time of <2 hours per day, the mean screen time was longer at 165 minutes (SD = 135). Accelerometer data (n = 130) showed low physical activity time (median 34 minutes [IQR = 29]). Only 18.5% of the total cohort met national recommended physical activity guidelines of 60 minutes per day. There were minimal ethnic differences. In conclusion, obese children/adolescents in this cohort had low levels of physical activity. The vast majority are not meeting national physical activity recommendations.

One of the key recommendations of the World Health Organization’s Report of the Commission on Ending Childhood Obesity is the promotion of physical activity^1^. Among OECD countries, New Zealand has the 3^rd^ highest prevalence of overweight and obesity in children and adults^2^. Recent research showed that obese New Zealand children and adolescents have suboptimal eating behaviours and a concerning prevalence of weight-related comorbidities^3^,^4^. The 2009 Clinical Guidelines for Weight Management in New Zealand Children and Young People recommend 60 minutes of moderate to vigorous-intensity aerobic physical activity per day, and less than two hours per day of screen time (televisions, computers and game consoles) outside of school hours^5^. However, although childhood obesity is a priority health issue in New Zealand, little is known about the levels of physical activity and sedentary behaviours of obese children in the country. The most comprehensive information comes from the National Survey of Children and Young People’s Physical Activity and Dietary Behaviours (2008/2009), where accelerometer data were collected from 1,812 individuals aged 5–24 years, alongside the collection of a Multi-media Activity Recall from Children and Adults (MARCA) data from a further 2,493 participants^6^. There was a recorded average of 114 minutes per day of moderate-intensity to very vigorous physical activity from accelerometer data in children aged 5–19 years (n = 1593). Of note, Māori, New Zealand’s indigenous population, were more active than their New Zealand European counterparts aged 5–19 years (mean 124 minutes [n = 285] vs. 113 minutes [n = 1193] respectively)^6^,^7^.

Most recently, the New Zealand Health Survey 2014/2015, which showed that 11% of children aged 2–14 years were obese, found that 45% of 2–14-year-old children watched at least two hours of television per day^8^. However, this survey is likely to have underestimated total screen time as it only captured time spent watching television^8^. The survey findings with respect to screen time mirrored the New Zealand ethnic and deprivation disparities in obesity. Māori children were 1.3 times and Pacific children 1.1 times more likely to watch over two hours of television per day compared with non-Māori and non-Pacific children^8^. Similarly, children living in the most deprived quintile of households were 1.5 times more likely to watch over 2 hours of television per day than children living in the least deprived quintile of households^8^.

Physical activity and screen time levels in overweight and obese New Zealand children referred to community-based intervention programmes have not been previously described, and there is a lack of information regarding potential differences between ethnic groups. The primary objective of this study was to describe the physical activity levels and sedentary behaviours of children and adolescents referred to a community-based obesity intervention programme (Whānau Pakari). Our secondary objective was to determine whether these outcomes differed between indigenous and non-indigenous children and adolescents.

## Methods

The Whānau Pakari programme^9^ was based in the Taranaki region, with a population of 23,139 children aged 0–15 years, of which 81% identify as New Zealand European, 28% as Māori, and 1% as other ethnicity^10^. Eligible participants were recruited between January 2012 and August 2014, were aged 5–16 years, and had a body mass index (BMI) ≥ 98^th^ percentile or BMI > 91^st^ percentile with weight-related comorbidities^11^.

Ethics approval was granted by the New Zealand Health and Disability Ethics Committee (CEN/11/09/054). Written and verbal informed consents were obtained from all participants or their guardians. This study was performed in accordance with all appropriate institutional and international guidelines and regulations for medical research, in line with the principles of the Declaration of Helsinki. Trial registration was with the Australian New Zealand Clinical Trials Registry (ANZCTR: 12611000862943).

### Study parameters

Each participant underwent a baseline assessment at home, where a number of assessments were undertaken including anthropometric measurements.

BMI percentile and BMI standard deviation score (SDS) were calculated using UK Cole normative data^12^ with the KIGS auxology software (Pfizer Endocrine Care TM). Socioeconomic deprivation was measured at the household level using the NZ Deprivation Index 2006^13^. This area level deprivation index, which is derived from national census data, is a well-validated measure of socioeconomic deprivation in New Zealand^13^.

Parents were asked to report their child’s/adolescent’s physical activity using the children’s physical activity questionnaire (C-PAQ)^14^. If children were ≥11 years of age, they were invited to participate in the questionnaire completion, with both participating in the final answers. The C-PAQ was chosen for the whole cohort due to the wide age range of participants, and lack of access to multimedia recall questionnaires within the home. Whilst all methods involving self report of physical activity have inherent associated error/bias, it was decided this questionnaire was most appropriate for this study population. The C-PAQ measures mode, frequency, and duration of physical and sedentary activities across all domains over the past 7 days. The C-PAQ has a validity of 0.42 (p = 0.04) and reliability of 0.39 (p < 0.05) for moderate and vigorous physical activity assessed by questionnaire^14^. It has been noted the low values may be due to the proxy-report nature of the questionnaire for younger children.

Apart from sports, duration of leisure activities (e.g. walking or skateboarding) were also recorded. Total non-school sedentary time was calculated from the questionnaire, and included activities such as art and craft, homework, listening to music, travelling in car/bus, reading, watching television/videos, and using the computer. Total screen time included all time using computer/internet/devices, watching television/videos, and playing computer games during a day outside of school hours. Time specifically watching television and videos was recorded for comparison with the New Zealand Health Survey.

Fitness was assessed within 8 weeks of initial baseline assessment using the 550-m walk/run test, which is a valid field test of cardiorespiratory fitness in children^15^.

Five days of ActiGraph wGT3X-BT (ActiGraph, Pensacola, Florida, USA) accelerometer wear (3 weekdays and 2 weekend days) were requested for each participant, aiming for an estimated reliability of 0.80 in children and 0.70 in adolescents^16^. A valid day was defined as ≥480 minutes of wear time per day^17^. Data were included if a minimum of 2 valid weekdays and 1 valid weekend day were obtained; reliability for 3.0 days of measurement overall was 0.7 in a large population-based study of 11-year-olds^17^. These levels of reliability have been questioned in more recent studies^18^, but longer wear time was not practical due to resource limitations. Epoch time was set to 60 seconds and cut-off time to 60 minutes. Non-wear time was defined as 60 minutes of consecutive zeros. Threshold values for accelerometer counts (counts/minute) were ≤99 for sedentary behaviour, ≤1951 for light (metabolic equivalent [MET] < 3.0), ≤5724 for moderate (MET 3.0–5.99), and ≤9498 for vigorous (MET 6.0–8.99) physical activity^19^. The Freedson adult cutoffs were used in order to undertake comparisons with national survey data^7^.

### Data analyses

Selected comparisons were made with 2012/2013 New Zealand Health Survey (NZHS) data regarding television use^20^. This survey best reflected the recruitment period for this cohort. We compared the data from our participants aged 5–14 years with that collected at the 2012/2013 NZHS, making these comparisons both with the NZHS national data and the dataset limited to study participants living in the Taranaki region. The NZHS included a random sample of predominantly non-obese children aged 5–14 years (n = 4,699) with data collected from their caregivers^20^. Selected comparisons were also made with the National Survey of Children and Young People’s Physical Activity and Dietary Behaviours in New Zealand (2008/2009)^6^. This nationally representative sample included 2,503 participants aged 5–24 years, of which 13% were identified as obese. Accelerometer data were attained for 1,812 of the participants^6^. An ActiGraph accelerometer was used, with light intensity physical activity represented as <3.0 METs, moderate intensity 3.0–5.99 METs, and vigorous intensity ≥6.0 METs^7^. 550-m walk/run data were compared with 2003 New Zealand data in 206 children aged 10–14 years, of which 7% of girls and 12% of boys were classed as obese^21^.

Categorical variables were compared between sexes using chi-square tests. Data were compared between ethnic groups using general linear or logistic regression models, adjusting for age, sex, and socioeconomic deprivation. Whānau Pakari data were compared to national survey data using 2-sample t-tests and 2-sample Poisson rate tests. Accelerometer and C-PAQ data were compared with paired t-tests and Cohen’s kappa coefficient. Data were analysed in Minitab (v.16, Pennsylvania State University, State College, PA, USA) and SAS v.9.4 (SAS Institute, Cary, NC, USA). All statistical tests were two-tailed with a significance level maintained at p < 0.05.

## Results

### Demographics and body mass index

Enrolled participants (n = 239) were a mean of 10.7 years (range 4.8–16.8 years), 52% were female, and with a BMI SDS of 3.09 (SD = 0.60, range 1.52–5.34 SDS). Participants were predominantly of Māori (45%) or NZ European (45%) ethnicity, with the remainder being of Pacific (3%), Asian (3%), or other (4%) ethnicities. Twenty-nine percent resided in households that were among the most deprived quintile of New Zealand households (compared with 15% of the total population of Taranaki)^13^,^22^,^23^. Of the 239 participants, nine (4%) had a BMI at the 98^th^ percentile, and 224 (94%) had a BMI > 98^th^ percentile on entry. Six (2%) participants entered with a BMI between the 91^st^ and 98^th^ percentile with weight-related comorbidities.

### Perceived physical activity and sedentary behaviours

234 participants completed the C-PAQ (98.3%), and their levels of physical activity and sedentary behaviours are shown in Table 1.

Our cohort reported they were more active in the weekdays compared with the weekends (p < 0.0001; Table 1), but this was not associated with an increase in overall weekend screen time. Nearly half of the participants (n = 111, 46%) had a television or computer in the bedroom^4^. Whilst 44% (n = 99) of our cohort met Ministry of Health screen time guidelines of less than two hours outside of school time (Table 1), the mean screen time was considerably longer at 165 minutes (SD = 135), with 34% with high screen time (≥3 hours per day).

Our cohort reported a daily mean time spent in sport activities of 30 minutes (median 20 minutes; Table 1), which is comparable to the figure of 29 minutes from the national survey of children and young people with a notably wider age range of 5–24 years^6^. In comparison to the New Zealand Health Survey national data, our cohort watched less television per weekday (means 1.5 vs. 1.9 hours; p = 0.001), but more throughout the whole weekend (means 5.0 vs. 3.9 hours; p = 0.004) (Table 2).

### Actual physical activity (accelerometer)

The accelerometer data collected from the 130 participants achieved valid wear for 2 (n = 63, 26%), 3 (n = 48, 20%), 4 (n = 12, 5%), and 5 weekdays (n = 8, 3%) and 1 (n = 63, 26%) or 2 weekend days (n = 75, 31%). Participants who had valid accelerometer wear were of similar age, BMI SDS, and level of deprivation as those who refused to wear or had non-valid wear. However, valid accelerometer wear was collected on a larger proportion of males than females (62% vs. 47%; p = 0.019). Reported total activity was also no different between the two groups. The accelerometer data for the 130 participants with valid wear are summarised in Table 3.

The mean time spent on moderate to vigorous-intensity physical activity was much lower in our cohort (39 minutes) than among 5 to 24 year olds in the national survey (105 minutes; p < 0.0001)^6^. Only 18.5% of our participants met the physical activity guidelines (Table 3), compared with 67.4% in the national survey (p < 0.0001)^6^. Of note, the likelihood of meeting these guidelines decreased with increasing age (p = 0.009) as observed in the national survey^6^, with a more marked reduction in physical activity levels among older children during weekends (p = 0.007). Although the proportion of participants found to meet physical activity guidelines was identical from accelerometer and C-PAQ data (Table 4), there was poor agreement between the two assessments (kappa = 0.13 [95% CI −0.06, 0.33]).

Interestingly, total reported C-PAQ activity levels (“lifestyle” to “very vigorous” physical activity) were lower than total activity measured with accelerometers (mean−42 minutes; p < 0.0001).

### Fitness

Participants (n = 164) performed the 550-m run/walk test in a mean time of 4.0 minutes (SD = 0.9), with the same average observed in both boys and girls. In comparison, 2003 New Zealand school population data for 206 10–14-year-olds (predominantly of normal weight) showed a mean speed of 3.3 m/s for boys and 3.1 m/s for girls^21^ vs. 2.5 and 2.4 m/s respectively, in our cohort.

### Ethnic Comparisons

When accounting for age, sex, and level of deprivation, the physical activity levels and sedentary behaviours were mostly similar between Māori and NZ European (Table 4). Although more Māori had a television or computer in the bedroom (54% vs. 40%; p = 0.034), screen time per day was similar in both groups (Table 4).

There were also no differences between Māori and NZ Europeans in the 550-m walk/run test (means 3.9 vs. 3.8 minutes respectively; p = 0.51). However, valid accelerometer wear was collected on a larger proportion of NZ Europeans than Māori (63% vs. 48%; p = 0.024). Notably, both Māori and NZ Europeans’ reported lower total activity compared with actual total activity levels, by a mean of 36 (p = 0.006) and 48 (p < 0.0001) minutes per day, respectively.

## Discussion

Our key findings were that obese children and adolescents in this cohort had low levels of physical activity, and the vast majority were not meeting physical activity national recommendations. Although over 40% of the cohort met the national recommended screen time of <2 hours per day, a third had high screen time ≥3 hours per day. The differences found between actual physical activity and sedentary activity highlight the value of utilising wearable devices to determine true levels of physical activity, despite the practical challenges. However, questionnaires and objective measures capture different aspects of physical activity, and questionnaires are unable to capture all body movements in the same way an accelerometer is able to^24^. Whilst physical activity estimates may be expressed in the same metrics, they are not conceptually equivalent. Therefore, the only comparison made between self-report and accelerometer data in this paper was total activity per day.

Even when objectively assessed, actual physical activity (moderate to very vigorous) in this cohort of obese children and adolescents was very low compared with predominantly non-obese counterparts from national survey data, with only 18.5% meeting physical activity guidelines. A limitation is that there are no national survey data for physical activity more recent than 2008/2009. Given there has been a reported increase in the proportion of 5–14-year-old predominantly non-obese children watching more than two hours of television or videos per day from 2002 to 2014/15 (27% per weekday and 40% per weekend day in 2002 to 45% per day in 2014/2015 [recent data 2–14-year-olds])^8^,^25^, it would be reasonable to expect that physical activity may have reduced in these counterparts over this time. This has been shown in a previous study of New Zealand children aged 10–14 years, where 550-m run times declined between 1991 and 2003^21^. More recent international literature does provide insight into normative data of simple step output for children. A review published in 2011 showed that, among children aged 6-11 years, it is reasonable to expect boys to average 12,000 to 16,000 steps per day, and girls to average 10,000 to 13,000 steps per day^26^. There is a steady decrease in number of steps per day throughout adolescence, with 8,000–9,000 steps per day observed in 18-year-olds^26^. The average steps per day in our cohort were below these figures, at 7,352 steps per day.

Weekend activity was less than weekday activity in our cohort, characterised by increased screen time compared with predominantly non-obese counterparts. Reduced weekend activity was consistent with a previous international study in British primary school children, which showed greater physical activity in weekdays (mean 13,827 steps) compared with weekends (mean 10,334 steps; p < 0.001)^27^.

A strength of this study was the high participation rate from Māori and those from the most deprived households, which allowed for comparison between indigenous and non-indigenous groups. Overall, we observed similar physical activity levels in Māori and NZE children and adolescents. This is important, as it was found that Māori in the 2002 National Children’s Nutrition Survey reported to be doing more overall activity than NZE children based on questionnaires^28^. The finding that more Māori children and young people had a television, computer or device in their bedroom is noteworthy, given previously reported findings in this cohort that a larger proportion of children with a device in their bedroom reported having difficulty getting to sleep (39% vs. 26%; p = 0.03)^4^.

Our study has some limitations. Comparisons with the National Survey of Children and Young People’s Physical Activity and Dietary Behaviours need to be interpreted with caution, given the wider age range in the survey (5–24 years), and the time lapse since the data were collected (2008/2009). Given that with increasing age there is a reduction in physical activity^6^, it is likely that the differences between our cohort and children of corresponding ages in this group were actually greater. Similarly, there were disparities with age range and a large time lapse with school population data for 550-m walk/run^21^. However, the main limitation of this study was the poor compliance with accelerometer wear, leaving only 58% of our total cohort with valid data. While the only differences between those with valid wear and the total cohort were related to ethnicity and sex, it does mean that findings relating to Māori need to be interpreted with caution. Technical limitations include the use of a 60-second Epoch which is likely to underestimate moderate to vigorous physical activity in children and adolescents, as it occurs in short intermittent bursts^29^. Whilst Freedson adult cutoffs were used in order to undertake comparisons with national data, it is acknowledged that Evenson cut-points are the most accurate in youth, and therefore, this would also affect the accuracy of physical activity data^30^.

Many methodologies exist for optimal accelerometer use. Some advocate for a 7-day monitoring protocol to achieve reliable estimates that mitigate for weekday and weekend variability^16^. In one study with children, a minimum duration of 6 hours for 7–9 days of monitoring (with 1 weekend day minimum) was required to achieve 80% reliability^18^. However, compliance is affected as monitoring extends, therefore the authors recommend an optimal balance between participant retention and achieving good reliability^18^. Given limited resource, this study aimed for a reliability of 0.7 with a minimum wear time of 480 minutes per day, based on a large field study of 11-year-olds^17^. Some studies are now including accelerometer data for all complete hours of wear time, arguing this captures participants less “compliant” with accelerometer use, and therefore limiting bias^31^.

In conclusion, this study shows that obese children and adolescents in the Taranaki region of New Zealand had low levels of physical activity. The majority are not meeting screen time or physical activity national recommendations, and this needs to be addressed in any future education programmes. Accelerometer data indicated no differences in physical activity levels in Māori and NZE, highlighting the need to address low levels of physical activity in all obese children and adolescents. As well as overall recommendations regarding increased physical activity and reduced screen time, targeted interventions amongst this population, including removing televisions and devices from bedrooms, and encouraging increased weekend physical activity are warranted.

## Additional Information

**How to cite this article**: Anderson, Y. C. *et al*. Physical activity is low in obese New Zealand children and adolescents. *Sci. Rep.* **7**, 41822; doi: 10.1038/srep41822 (2017).

**Publisher's note:** Springer Nature remains neutral with regard to jurisdictional claims in published maps and institutional affiliations.

## Figures and Tables

**Table 1:** Physical activity levels and sedentary behaviours (child self-report or parent proxy-report) using the C-PAQ questionnaire, among children and adolescents (n = 234) enrolled in Whānau Pakari.

Total activity weekday (min)	88 [50, 141]
Total activity weekend day (min)	45 [0, 104]
Total activity per day for whole week (min)	79 [44, 132]
Leisure activity per day (min)	34 [17, 79]
School-related activity per day (min)	12 [0, 30]
Sport activity per day (min)	20 [6, 43]
Non- school sedentary time per day (min)	282 [163, 420]
Adequate physical activity^a^	45 (19.3%)
Screen time per weekday (min)	120 [60, 198]
Screen time per weekend day (min)	150 [49, 300]
Total screen time per day for whole week (min)^b^	129 [76, 223]
Screen time below 2 hours^c^	103 (44%)

Data are medians [Q_1_, Q_3_] or n (%).

^a^>60 minutes of moderate-intensity to very vigorous physical activity per day as per Ministry of Health recommendations.

^b^Screen time at school not included.

^c^As per New Zealand Ministry of Health recommendation^5^.

**Table 2:** Time spent watching television (child self-report or parent proxy-report) among children and adolescents enrolled in Whānau Pakari. Data only covers respondents aged 5 to 14 years to allow comparison with the New Zealand Health Survey 2012–2013^20^.

	Whānau Pakari	New Zealand Health Survey	
		National	Taranaki
n	169	2,717	127
Time per weekday (h)	1.5 (1.5)	1.9 (2.9)**	2.2 (2.6)**
n	143	2,692	125
Total weekend (h)	5.0 (4.2)	3.9 (3.8)**	4.8 (6.4)

Data are means (standard deviations). **p < 0.01 for comparisons with Whānau Pakari.

**Table 3:** Accelerometer data for children and adolescents enrolled in Whānau Pakari who had valid wear (n = 130), and their respective C-PAQ activity where appropriate.

	Accelerometer	C-PAQ
Non-school sedentary time weekday (min)	341 [276, 414]	—
Non-school sedentary time weekend day (min)	352 [261, 436]	—
Total non-school sedentary time per day for whole week (min)	340 [274, 405]	269 [165, 387]*
Steps per weekday	7412 [5921, 8949]	—
Steps per weekend day	6351 [4067, 8611]	—
Total steps per day for whole week	6789 [5869, 8782]	—
Energy expenditure per day (kcal)	424 [275, 574]	—
Total activity per day (min)	130 [96, 165]	79 [43, 131]****
Moderate-intensity to very vigorous physical activity per day (min)	34 [24, 53]	—
Adequate physical activity^a^	24 (18.5%)	24 (18.5%)

Data are medians [Q_1_, Q_3_] or n (%). *p < 0.05 and ****p < 0.0001 for comparison between accelerometer and C-PAQ data.

^a^>60 minutes of moderate-intensity to very vigorous physical activity per day as per Ministry of Health recommendations.

**Table 4:** Accelerometer data, as well as physical activity levels and sedentary behaviours self-reported or reported by parents using the C-PAQ questionnaire among children and adolescents enrolled at Whānau Pakari.

		Māori	NZ Europeans	p-value
C-PAQ	n	106	106	
	Total activity per day (min)	83 [52, 135]	79 [39, 127]	0.67
	Sport activity per day (min)	29 [9, 51]	16 [1, 34]	0.10
	Total non-school sedentary time per day for whole week (min)	269 [145, 380]	259 [171, 439]	0.92
	Total screen time per day for whole week (min)^a^	107 [60, 199]	143 [86, 247]	0.59
	Screen time below 2 hours^b^	54 (51%)	42 (40%)	0.13
Accelerometer	n	56	70	
	Total sedentary time per day for whole week (min)	344 [272, 417]	340 [277, 412]	0.33
	Total steps per day	7140 [5933, 9169]	6789 [5852, 8536]	0.95
	Total activity per day (min)	135 [93, 175]	124 [101, 164]	0.31
	Moderate-intensity to very vigorous physical activity per day (min)	33 [23, 56]	34 [23, 51]	0.40
	Adequate physical activity^c^	13 (23%)	9 (13%)	0.18

Data are medians [Q_1_, Q_3_] or n (%).

^a^Screen time at school not included.

^b^As per New Zealand Ministry of Health recommendation^5^.

^c^>60 minutes of moderate-intensity to very vigorous physical activity per day as per Ministry of Health recommendation.
